# Isolation and Environmental Detection of *Balamuthia mandrillaris* in Isfahan, Iran

**DOI:** 10.1111/1758-2229.70156

**Published:** 2025-09-01

**Authors:** Sara Lesani, Soroush Mirzania, Abbasali Eskandarian

**Affiliations:** ^1^ Department of Parasitology and Mycology School of Medicine, Isfahan University of Medical Sciences Isfahan Iran

**Keywords:** *Balamuthia mandrillaris*, cultural detection, emerging pathogens, free‐living amoebae, molecular diagnostics

## Abstract

*Balamuthia mandrillaris* is a free‐living opportunistic amoeba known to cause fatal encephalitis. Despite its global environmental presence, data on its distribution in Iran and comparative detection methods remain scarce. This study aimed to investigate the occurrence of *B. mandrillaris* in various environmental sources in Isfahan, Iran, and to evaluate the performance of culture‐based and molecular detection approaches. A total of 96 environmental samples (33 soil, 31 dust, and 32 water) were collected from different locations across Isfahan. All samples were examined in parallel using culture methods and PCR amplification targeting the mitochondrial 16S rDNA gene. Selected PCR‐positive samples underwent sequencing to confirm species identity. The overall detection rate of *B. mandrillaris* was 15.6% using both culture and molecular techniques, with soil samples showing the highest prevalence (24.2% by molecular and 21.2% by culture). Molecular and cultural methods displayed complementary but matrix‐dependent detection patterns, with a substantial agreement between methods (*κ* = 0.72). This study confirms the presence of *B. mandrillaris* in various environmental sources in Isfahan, highlighting soil as a potential reservoir. The findings emphasise the importance of combined molecular and culture‐based approaches and suggest raising public health awareness, even in regions without reported clinical cases.

## Graphical abstract

This study investigates the presence of *Balamuthia mandrillaris* in environmental sources from Isfahan, Iran, and compares cultural and molecular detection methods. The findings highlight matrix‐dependent detection patterns and report an overall prevalence of 15.6%.

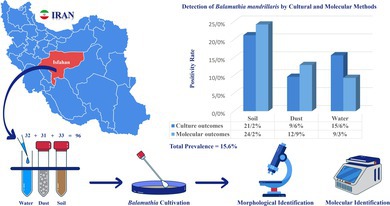

## 1 Introduction

Free‐living amoebae are ubiquitous microorganisms that inhabit diverse environmental niches surrounding human settlements, including soil, water, and dust (Meshjel 2024). Whilst several species within this group are known pathogens, including *Acanthamoeba*, *Sappinia*, and *Naegleria*, *Balamuthia mandrillaris* has emerged as a particularly concerning opportunistic pathogen due to its ability to cause severe infections in both immunocompromised and immunocompetent hosts across a wide geographic range (Fan et al. 2024; Mungroo et al. 2022).

*B. mandrillaris* was first isolated in 1986 from a mandrill baboon that succumbed to encephalitis at the San Diego Zoo Wildlife Alliance. Since this initial discovery, the organism has been identified as a pathogen capable of infecting various mammals, including humans of all ages and immune statuses. The first human case was documented in 1990, and subsequent infections have predominantly been reported in South and North America, particularly in regions characterised by warm, desert, or tropical climates. However, the organism's remarkable adaptability enables it to survive in diverse environmental conditions (Bhosale and Parija 2021).

Morphologically, *B. mandrillaris* bears a striking resemblance to *Acanthamoeba*, especially when observed in tissue sections under light microscopy. The organism exhibits a dimorphic life cycle consisting of two distinct stages. The trophozoite stage is characterised by an irregular shape, ranging from 12 to 60 μm with a mean size of approximately 30 μm (Cope et al. 2019). Whilst typically uninucleate, binucleate forms have been observed, with the nucleus containing a prominent, centrally positioned nucleolus. The cyst stage is predominantly spherical and uninucleate, measuring 12–30 μm with a mean diameter of 15 μm. These cysts exhibit a triple‐layered wall structure, comprising an undulating outer ectocyst, a spherical inner endocyst, and an intermediate amorphous fibrillar mesocyst, as confirmed by transmission electron microscopy. However, only the ectocyst and endocyst are typically discernible under light microscopy (Siddiqui and Khan 2015).

The pathogenesis of *B. mandrillaris* infection involves multiple entry routes into the human body, including skin lesions, neuroepithelia, and the olfactory and pulmonary systems (Siddiqui and Khan 2015). Following entry, the organism reaches the central nervous system through hematogenous spread, though the precise mechanism by which it traverses the blood–brain barrier remains unclear. This gap in understanding stands in contrast to the better‐characterised mechanisms of *Acanthamoeba*, which employs a combination of cell adhesion, protease secretion, and host inflammatory responses to breach the blood–brain barrier (Jayasekera et al. 2004; Inglis et al. 2003).

*Balamuthia* amoebic encephalitis (BAE), the primary manifestation of infection, presents with symptoms that overlap considerably with viral and bacterial meningitis, including headaches, nausea, neck stiffness, fever, and photophobia. As the infection progresses, patients may develop more severe neurological manifestations such as personality changes, aphasia, confusion, and lethargy, ultimately leading to death in most cases (Ono et al. 2024). The challenge of ante‐mortem diagnosis is significant, with most cases being identified through post‐mortem histological examination of infected tissue samples (Khurana et al. 2015).

Despite the global health implications of *B. mandrillaris*, significant gaps remain in our understanding of its geographic distribution, particularly in the Middle East. Previous environmental surveys in Iran have successfully isolated *B. mandrillaris* from dust samples in Tehran (Niyyati et al. 2009), soil samples in East Azerbaijan (Niyyati et al. 2016), and hot springs in northern Iran (Latifi et al. 2016), demonstrating its presence in diverse environmental niches. However, the distribution pattern of this pathogen in central Iran, particularly in Isfahan province, remains unexplored. This knowledge gap is particularly concerning given the organism's ability to thrive in various environmental conditions and its demonstrated presence in Iranian ecosystems. In this study, we conducted a comprehensive survey of 96 environmental samples from Isfahan, employing both cultural and molecular methods, to expand our understanding of *B. mandrillaris* distribution.

## 2 Materials and Methods

### 2.1 Study Design and Sample Collection

We conducted a systematic environmental survey across Isfahan province, Iran. The urban region was stratified into four sampling zones using randomised block design. We collected 96 environmental samples: soil (*n* = 33, 15 g in sterile containers), water (*n* = 32, 1 L in sterilised borosilicate bottles), and surface dust (*n* = 31, using sterile rayon swabs).

### 2.2 Culture Media and Enrichment

Non‐nutrient agar media were prepared with Page's modified amoeba saline (2.5 mM NaCl, 1 mM KH_2_PO_4_, 0.5 mM Na_2_HPO_4_, 0.4 mM CaCl_2_·6H_2_O, 0.02 mM MgSO_4_·7H_2_O, pH 6.9). The medium was supplemented with heat‐killed *Escherichia coli* ATCC 25922 (400 μL/plate at OD600 = 2.0) as a nutrient source. Quality control was performed using *B. mandrillaris* CDC: V039 as reference strain (Mündler 2016).

### 2.3 Sample Processing

Water samples were processed through 8‐μm cellulose nitrate membrane filters under aseptic conditions. Soil samples were homogenised and suspended 1:1 (w/v) in sterile phosphate‐buffered saline. Dust samples were eluted in 15 mL sterile isotonic solution. From each processed sample, 500 μL was inoculated onto culture plates. All plates were sealed and incubated at 25°C ± 1°C for 60 days with daily monitoring.

### 2.4 Morphological Identification

**FIGURE 1:**
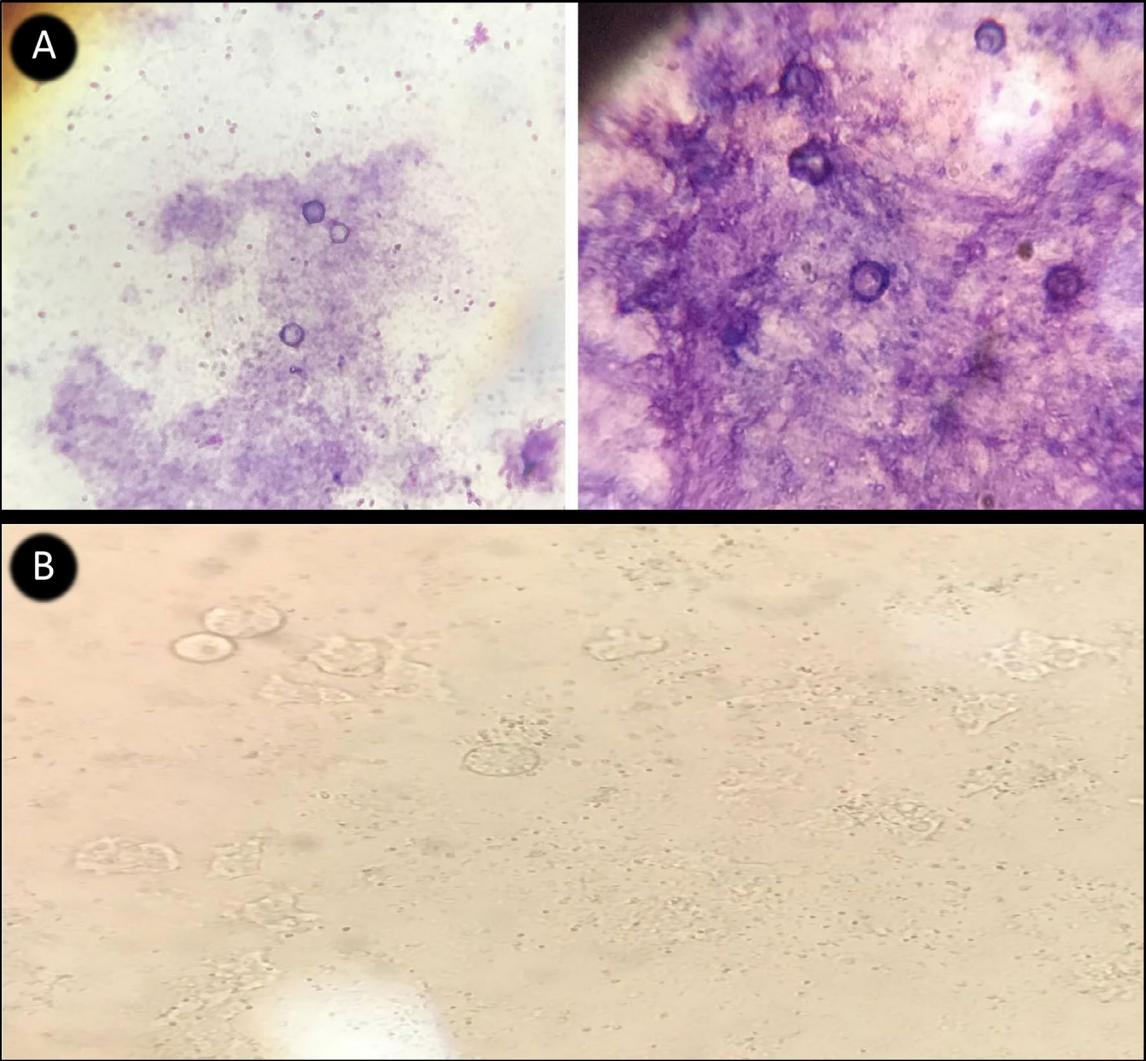
Giemsa‐stained cysts and trophozoites of *Balamuthia mandrillaris* isolated from environmental samples in Isfahan, Iran. (A) Cysts showing characteristic double‐walled structure (12–30 μm in diameter) with a wavy ectocyst and spherical endocyst morphology, typical of *B. mandrillaris* (Magnification: Left: 100×; Right: 400×). (B) Trophozoites of *B. mandrillaris* observed under phase‐contrast microscopy, exhibiting irregular amoeboid morphology with prominent nucleoli.

Cultures were examined daily using phase‐contrast inverted microscopy (100×, 400×). Positive samples showing characteristic trophozoites (12–60 μm) and double‐walled cysts (12–30 μm) were documented using calibrated photomicrography. Selected isolates were stained with Giemsa for detailed morphological characterisation (Figure 1).

### 2.5 Isolation of Amoeba From Culture Plates

When characteristic trophozoites and cysts of *B. mandrillaris* were observed under the microscope, a sterile micropipette was used to carefully aspirate the amoeba‐rich liquid layer from the agar surface. This suspension was then centrifuged at 1500 rpm for 10 min to concentrate the cells, and the pellet was washed twice with sterile phosphate‐buffered saline (PBS). The purified pellet was subsequently used for DNA extraction and molecular confirmation.

## 3 Molecular Analysis

### 3.1 DNA Extraction and Quality Assessment

Amoebae were harvested using sterile Page's saline and concentrated by centrifugation (5000 RPM, 15 min). Genomic DNA was extracted using phenol‐chloroform‐isoamyl alcohol methodology. DNA quality and quantity were assessed spectrophotometrically (A260/A280 ratio) and by agarose gel electrophoresis.

### 3.2 PCR Amplification

The mitochondrial 16S rDNA gene was amplified using validated primers BalspecF (5′‐CGCATGTATGAAAGAAGACCA‐3′) and BalspecR (5′‐CCCCTTTTAACTCTAGTCATATAGT‐3′). PCR reactions (25 μL) contained: 12.5 μL 2× master mix, 1.5 μL each primer (10 μM), 2 μL template DNA, 7.5 μL nuclease‐free water. Cycling conditions: initial denaturation at 95°C (5 min); 35 cycles of 94°C (30s), 60°C (45 s), 72°C (60s); final extension at 72°C (10 min) (Booton et al. 2003).

### 3.3 Gel Electrophoresis and Sequencing

PCR products were analysed by electrophoresis on 1.5% agarose gels stained with DNA‐safe dye. Positive samples with a 230‐bp amplicon were purified and sent to Macrogen Inc. (South Korea) for sequencing. Sequencing results were compared against NCBI database records using BLAST to confirm species.

## 4 Statistical Analysis

Statistical analyses were conducted using GraphPad Prism version 9.0 (GraphPad Software, USA). Chi‐square tests with Bonferroni correction for multiple comparisons were employed to assess differences in positivity rates amongst sample types. Relative risk ratios were calculated to compare contamination rates between different environmental sources. Diagnostic performance metrics including sensitivity, specificity, positive predictive value (PPV), and negative predictive value (NPV) were calculated with 95% confidence intervals using the Wilson score method. The degree of agreement between morphological and molecular detection methods was evaluated using Cohen's kappa coefficient. Statistical significance was set at *p* < 0.05 for all analyses.

## 5 Results

Our analysis of 96 environmental samples from Isfahan revealed an overall positivity rate of 15.6% (15/96) for both culture and molecular detection of *B. mandrillaris*. In molecular analysis, soil samples showed the highest detection rate at 24.2% (8/33), followed by dust at 12.9% (4/31), and water samples at 9.3% (3/32). Culture methods revealed slightly different patterns, with soil at 21.2% (7/33), water at 15.6% (5/32), and dust at 9.6% (3/31) (Table 1). Soil samples consistently showed significantly higher positivity rates compared to both dust (*p* < 0.05) and water samples (*p* < 0.01) across both methods.

**TABLE 1:** Comparison of culture and molecular methods for detection of *B. mandrillaris* in environmental samples from Isfahan, Iran.

Sample type	Total samples	Culture outcomes		Molecular outcomes		Mol+/Cul−	Mol−/Cul+
		Positive (*n*, %)	Negative (*n*, %)	Positive (*n*, %)	Negative (*n*, %)		
Soil	33	7 (21.2)	26 (78.8)	8 (24.2)	25 (75.7)	2	1
Dust	31	3 (9.6)	28 (90.3)	4 (12.9)	27 (87.1)	1	0
Water	32	5 (15.6)	27 (84.3)	3 (9.3)	29 (90.6)	2	4
Total	96	15 (15.6)	81 (84.3)	15 (15.6)	81 (84.3)	5	5

*Note:* Percentages may not sum to exactly 100% due to rounding to one decimal place.

The quality assessment of extracted DNA demonstrated high purity across all sample types. Mean A260/A280 ratios were 1.82 ± 0.24 (range: 1.4–2.1) for soil samples, 1.65 ± 0.18 (range: 1.28–1.89) for dust samples, and 1.54 ± 0.21 (range: 1.1–1.98) for water samples. DNA concentrations ranged from 1.1 to 4.3 μg/mL, providing sufficient template for reliable PCR amplification.

PCR amplification targeting the mitochondrial 16S rDNA gene yielded the expected 230‐bp product in all molecularly positive samples. Due to financial and logistical limitations, only 10 out of the 15 PCR‐positive samples were selected for sequencing, ensuring representation from each sample type. BLAST analysis of the obtained sequences showed 96% identity with registered reference sequences of *B. mandrillaris* in the GenBank database, confirming the molecular identity of the detected organisms.

Comparative analysis of morphological and molecular detection methods revealed a sensitivity of 66.7% (95% CI: 58.2%–75.2%) and specificity of 93.8% (95% CI: 88.9%–98.7%) for molecular detection. The positive predictive value was 75% (95% CI: 67.3%–82.7%), whilst the negative predictive value reached 91.4% (95% CI: 86.2%–96.6%). We observed 5 cases each of molecular‐positive/culture‐negative (Mol+/Cul−) and molecular‐negative/culture‐positive (Mol−/Cul+) results. The Cohen's kappa coefficient (*κ* = 0.72) indicated substantial agreement between the two detection methods.

Statistical analysis of environmental distribution patterns revealed significant differences in contamination rates across sample types (*χ* ^2^ = 7.82, d*f* = 2, *p* < 0.05). By molecular methods, soil samples demonstrated 2.6‐fold higher positivity compared to water samples (24.2% vs. 9.3%, *p* < 0.001) and 1.9‐fold higher than dust samples (24.2% vs. 12.9%, *p* < 0.05). Culture methods showed a different pattern, with soil samples at 21.2%, water at 15.6%, and dust at 9.6%.

## 6 Discussion

Our comprehensive analysis of *B. mandrillaris* distribution in urban environments reveals several critical findings with significant implications for public health surveillance and epidemiological understanding. The parallel detection rates of 15.6% by both cultural and molecular methods, whilst seemingly concordant, mask important methodological variations that deserve careful consideration.

The predominance of *B. mandrillaris* in soil samples (24.2% molecular, 21.2% cultural) compared to other environmental sources aligns with previous findings from California (Dunnebacke et al. 2004), Peru (Cabello‐Vílchez et al. 2014), Turkey (Aykur and Dagci 2023), and Japan (Yamanouchi et al. 2018), where soil has been identified as a primary reservoir. This is particularly significant in the Iranian context, where prior studies have demonstrated successful isolation of *B. mandrillaris* from environmental sources in only seven countries worldwide, including Iran. The previous isolation from dust in Tehran (Niyyati et al. 2009), hot springs in northern Iran (with 2 positives from 66 water samples) (Latifi et al. 2016), and our findings in Isfahan, coupled with the report from East Azerbaijan showing 5 positive samples out of 55 soil specimens (Niyyati et al. 2016), collectively strengthen the evidence for Iran as a significant geographical region for *B. mandrillaris* distribution. This is especially noteworthy given that despite the organism's presence in various environmental sources in Iran, no clinical cases have been reported in the country, suggesting potential environmental adaptations or unrecognised cases. However, our study uniquely demonstrates that detection methods can significantly influence observed distribution patterns. The higher molecular detection rate in soil samples, coupled with better cultural recovery from water samples (15.6% vs. 9.3%), suggests that environmental matrices may differentially affect organism viability and detection sensitivity. This methodological divergence has not been previously emphasised in environmental surveillance studies and carries important implications for sampling strategies, particularly given the rarity of environmental isolation events globally. The successful isolation from diverse environmental sources in Iran—including soil, dust, and hot springs—suggests that regional environmental conditions might play a crucial role in the organism's distribution and detection sensitivity across different matrices, emphasising the need for comprehensive surveillance approaches incorporating multiple sample types and detection methods.

The concordance analysis between detection methods (*κ* = 0.72) reveals both strengths and limitations of current surveillance approaches. The observation of equal numbers of discordant results (5 Mol+/Cul− and 5 Mol−/Cul+) challenges the traditional view of molecular methods as universally superior. The moderate sensitivity (66.7%) but high specificity (93.8%) of molecular detection suggests that a combined methodological approach might be optimal for environmental surveillance. These findings contrast with previous studies that typically relied on single detection methods, potentially underestimating true prevalence rates (Niyyati et al. 2009, 2016; Ahmad et al. 2011).

The spatial distribution of positive samples across urban environments raises important questions about human exposure risks. The significant variation in detection rates across sample types (*p* < 0.05) suggests that environmental factors influence *B. mandrillaris* persistence and distribution. This heterogeneous distribution pattern, particularly evident in soil samples, indicates that targeted surveillance of high‐risk environments might be more effective than broad‐spectrum sampling.

This study has several limitations that should be acknowledged. First, environmental sampling was conducted during a single seasonal period, which may not adequately capture the annual variability in the distribution and prevalence of *B. mandrillaris* in the region. Second, although molecular methods confirmed the presence of *B. mandrillaris* DNA, the viability of the organism could not be determined for samples that were PCR‐positive but culture‐negative, leaving the potential transmission risk unresolved. Third, due to financial and logistical constraints, sequencing was restricted to a subset of PCR‐positive samples, limiting the depth of molecular characterisation. Additionally, the use of Page's modified saline medium for culturing, whilst cost‐effective and compatible with laboratory protocols, may have influenced recovery rates compared to alternative media such as BM3 or BMI, which could offer higher sensitivity or selectivity for *B. mandrillaris* isolation.

## 7 Conclusions

Our study demonstrates the widespread presence of *B. mandrillaris* in environmental sources across Isfahan, Iran, with soil identified as the primary reservoir. The combined use of culture and molecular detection methods proved to be both complementary and effective, underscoring the importance of applying multiple diagnostic approaches for reliable environmental surveillance. Although no clinical cases have been reported in Iran to date, the confirmed presence of *B. mandrillaris* in soil, dust, and water samples highlights a potential public health concern and the need for increased clinical awareness and monitoring. These findings provide valuable insights into the ecology of *B. mandrillaris* and establish a foundation for future research on its environmental distribution and pathogenic potential.

## Author Contributions

**Sara Lesani:** data curation, formal analysis, investigation, visualization, writing – original draft. **Soroush Mirzania:** investigation, methodology, laboratory work. **Abbasali Eskandarian:** conceptualization, methodology, supervision, validation, writing – review and editing, project administration.

## Conflicts of Interest

The authors declare no conflicts of interest.

## Data Availability

The data that support the findings of this study are available on request from the corresponding author. The data are not publicly available due to privacy or ethical restrictions.
